# Public Health and Emigration

**Published:** 1922-05

**Authors:** G. T. K. Maurice

**Affiliations:** C.M.G., C.B.E.


### PUBLIC HEALTH AND EMIGRATION.

By COLONEL G. T. K. MAURICE, C.M.G., C.B.E.

On February 10 a deputation from the Royal Colonial Institute was received by Mr. Winston Churchill, with the object of urging the importance of emigration to the Over-Seas Empire as a remedy for unemployment in Great Britain. Great Britain has now a population greater in numbers than is desirable, and that is one of the causes of the unemployment now so disastrous. These are apparently the beliefs of the supporters of the deputation. The Colonial Secretary did not express an opinion on the relation between numbers and unemployment, but he cordially endorsed the idea of assisting numbers of the inhabitants of Great Britain to emigrate, and he foreshadowed a Bill and taxpayers' money to assist the flow.

Whether the policy be right or wrong, there is a public health factor that should be considered. The emigrants will not be of the worst of the population, for two reasons. Men and women who make up their minds to uproot themselves and to try for fortune in lands far away are of those who feel strong and capable, of the ambitious, of those who are willing to work, of the men of long vision, content with the prospect of hardship and toil so it ha in it hope of ultimate wealth; not necessarily great wealth, but an inc ease on what they have, and a rise to above the position of stagnant wage-getter. The feeble man, the man of no ambition, the slack, the sickly, the work-shy, he who is content to work to-day that he may idle and shirk to-morrow, and he who is happy so long as he receives he public charity of the dole or the private charity of the sentimental, do not set themselves to face the new. They have not the grit. The Dominions insist on selection, and choose of the best. Before Canada will admit a man, he must have a clean bill of health, a good character and at least £50 of savings, besides his ticket to his destination. If he have a wife with him, she also must be physically and morally of the better part of the population and have £25 to keep herself at starting. Though it is not to the point of public health, it is worth while to note that British taxpayers and ratepayers, collectively or as individuals, pay for the rearing and educating of these emigrants during the years when they are children and adolescents not able to earn their livelihood. Now they are to pay passage-money for many of them and send them, with at least £50 of capital, into another land, to another people, kin, it is true, who will have the advantage of them, free of all cost, just as they have attained to full wealth-earning capacity and during the time that capacity endures. Maybe Great Britain's expenditure is indirectly recouped.

It is a fact of evolution that, if any cause tends to advantage the survival of a certain variety of a species in a certain district, that variety, by steady increase, becomes the chief type of the species, and ultimately squeezes out other types by breeding its like; its favourable to survival variations. Now the variations enumerated that send a man seeking fortune and the peculiarities enumerated that keep a man at Home are many of them congenital characteristics, therefore inheritable. The tendency will be for the inhabitants of the over-sea Dominions to be bred from part of the best specimens of the people of the British Isles and to inherit their cha acteristics, and for the Home stock to be bred from the remainder of the best and all the worst and to inherit their characteristics. This, it appears certain, mus entail a steady deterioration of the average physique and capacity and character of the people of Great Britain. As an Empire question, this may be compensated by the upspringing of races beyond the seas as good or better than Britain ever bred. The steady deterioration foreshadowed may accelerate somewhat rapidly in this way. If the number of incapables rises, the weight of them will be heavier and heavier, on the comparatively few capables. Each wealth-producer will have to give a greater share of his earnings to help the incapables. The incapables more and more will have the most votes to count, and will freely use them to help themselves at the expense of the capables. Thus, the tendency of the capables to emigrate to other lands will be increased. In a steadily rising proportion they will leave to escape the burden.

Emigration would greatly assist the problem of unemployment if it were that the unemployable could be sent across the seas. It is, however, doubtful if many men of the sort Canada or Australia is willing to accept are unable to find work here. If that sort goes out of Great Britain, the going may relieve unemployment by leaving good men's places vacant for less good men. The price of relief will be heavy if it prove to be a feeble race at Home.

### THE CARE OF TONSIL AND ADENOID CASES.

The Council of the Laryngological Section of the Royal Society of Medicine has made the following suggestions with reference to operations for tonsils and adenoids:—

(1) That all clinics, whether at hospitals or schools, should be in the charge of surgeons with special experience of diseases of the nose, throat and ear, so that, *inter alia*, a wise selection may be made of cases requiring operation, and others not requiring operation may be appropriately treated; (2) That all patients requiring operations for tonsils and adenoids should have in-patient institutional treatment, and that a stay of at least 48 hours should be insisted on, and a further stay if thought advisable by the medical officer in charge; (3) That parents should be given printed instructions as regards the preparation of the patient for operation and of the room to which the patient will return; (4) That before the patient is admitted for operation inquiries should be made by a responsible authority as to the home conditions and circumstances, especially with reference to the presence of infectious disease; (5) That when the patient leaves the hospital printed instructions with regard to after-treatment should be given; (6) That anæsthetics should be given by anæsthetists with special experience of these operations; (7) That after the patient leaves the hospital with the printed instructions for after-treatment, arrangements should be made for the supervision of a qualified visiting nurse.

